# Effectiveness of sarolaner in the clinical management of furuncular myiasis in dogs naturally infested with *Dermatobia hominis* (Diptera: Cuterebridae)

**DOI:** 10.1186/s13071-021-04910-3

**Published:** 2021-08-13

**Authors:** Paula A. Andriotti, Clarissa P. Souza, Priscila C. Oliveira, Rodrigo C. Melo, Guilherme G. Verocai, Julio I. Fernandes

**Affiliations:** 1 Department of Animal Parasitology, Veterinary Institute, Federal Rural University of Rio de Janeiro, Seropédica, RJ Brazil; 2 Department of Veterinary Clinical Medicine, College of Veterinary Medicine, University of Illinois at Urbana-Champaign, Urbana, IL USA; 3 Small Animal Veterinarian, Paty de Alferes, RJ Brazil; 4 Department of Veterinary Pathobiology, College of Veterinary Medicine and Biomedical Sciences, Texas A&M University, College Station, TX USA; 5 Department of Veterinary Medicine and Surgery, Veterinary Institute, Federal Rural University of Rio de Janeiro, Seropédica, RJ Brazil

**Keywords:** *D. hominis*, Sarolaner, Treatment, Isoxazolines

## Abstract

**Background:**

The human botfly, *Dermatobia hominis*, is a common cause of furuncular myiasis in dogs in Latin America. Lesions can be single or multiple, each harboring an individual larva, presented as an erythematous nodule that causes pruritus and pain. Typical treatment consists of sedation for removal of larvae by surgical incision or manual pressure. Medications to kill the larva before its extraction can reduce inflammation and discomfort and provide a less traumatic larval removal. Isoxazolines are broad-spectrum ectoparasiticides with larvicidal activity previously reported in the treatment of screwworm myiasis in companion animals. The aim of this study was to evaluate the effectiveness of sarolaner as part of the clinical management of furuncular myiasis in dogs caused by *D. hominis* larvae.

**Methods:**

Ten short-haired mixed breed dogs naturally infested with *D. hominis* were enrolled. Clinical diagnosis was achieved by observation of skin nodules and visualization of larval motility through the lesion orifice. Sarolaner was administered at manufacturer recommended dose for fleas and ticks. Lesions were reexamined 24 h post-treatment and assessed for viability of larvae. Larvae were removed by digital compression and identified as *D. hominis.*

**Results:**

Seventy-five *D. hominis* larvae were retrieved from ten dogs. No live larvae were observed, demonstrating 100% larvicidal efficacy of sarolaner. Skin lesions were healed 30 days post-treatment and new lesions were not observed.

**Conclusions:**

Sarolaner seems to be effective as larvicidal treatment for dogs with furuncular myiasis, reducing discomfort caused by the presence of the larva in the skin and facilitating its safe removal.

**Graphical abstract:**

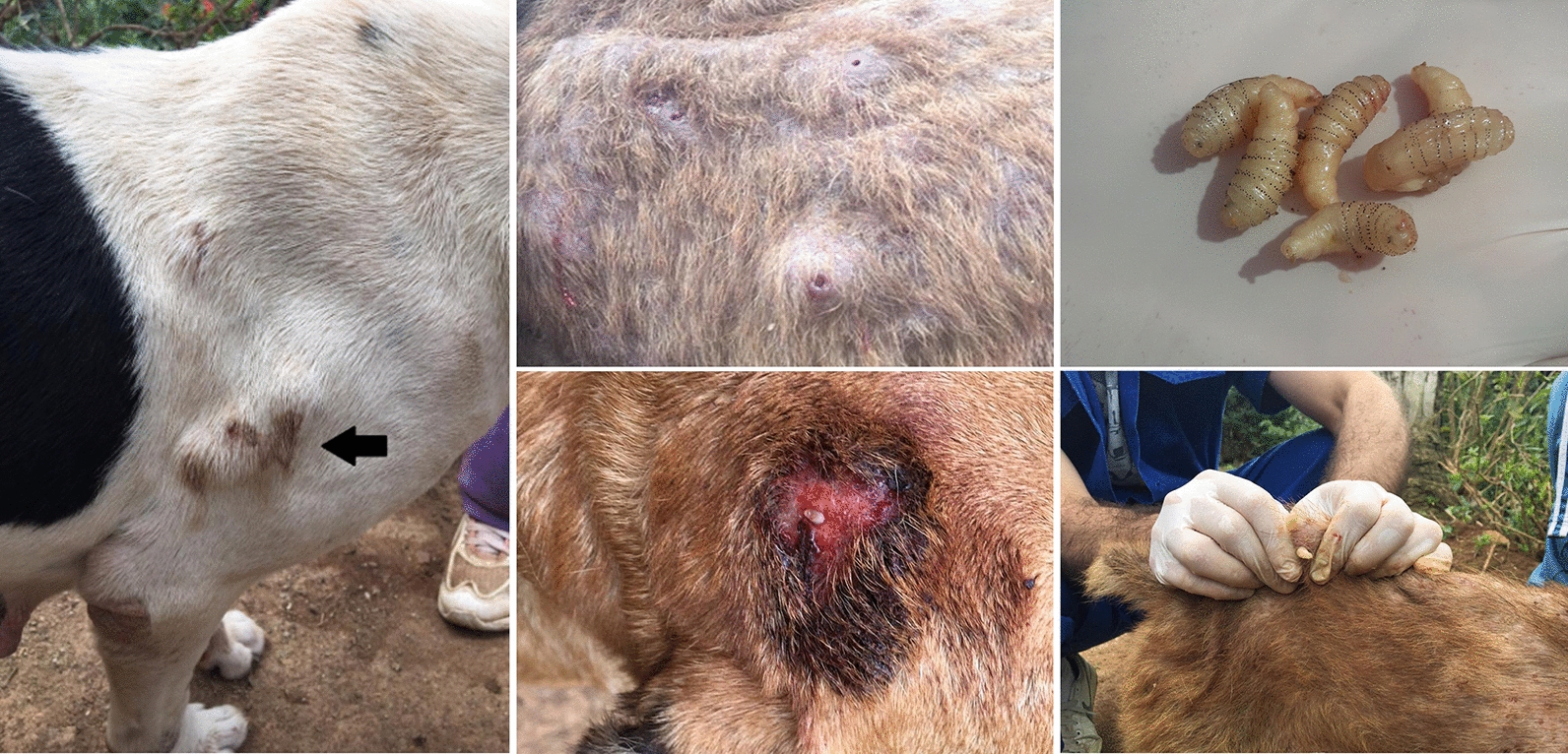

Myiasis is the infestation of a vertebrate host by dipteran larvae, usually known as maggots or bots. When humans and different animal species develop furuncular lesions as a result of skin penetration by an individual larva of a primary myiasis-causing fly, it is called furuncular myiasis. The human botfly, *Dermatobia hominis*, is a common cause of furuncular myiasis in livestock and relatively common in companion animals and humans across Latin America, whereas *Cuterebra* sp. is commonly reported causing furuncular myiasis in companion animals, rodents and lagomorphs in North America [1].

Furuncular myiasis lesions can be single or multiple, each harboring a single larva. Clinically, the lesions present as erythematous nodules with an orifice that exudates serosanguineous discharge and where the posterior end of the larva can be seen [2]. The infested dogs experience pruritus and pain from the various rows of robust, outer spines of the larva within the skin [3]. The usual treatment for furuncular myiasis consists of complete removal of larva by a surgical incision and insertion of an instrument to pull the larva out or manual pressure preceded by lidocaine injection around the lesions. Ideally, the dogs should be sedated for adequate removal of larvae and larval products. The use of medications to kill the larvae before their extraction can be an alternative to help reducing inflammation, to prevent discomfort and to allow for a less traumatic larval removal in infested dogs, especially when multiple lesions exist [3].

The isoxazolines are a novel class of ectoparasiticides with inhibitory activity on glutamate and gamma aminobutyric acid-gated chloride channels located in nervous system of invertebrates [4]. Isoxazoline compounds such as fluralaner, afoxolaner, sarolaner and lotilaner are labeled for the treatment and control of fleas and ticks in dogs and cats. Additionally, effective off-label use of several isoxazolines has been reported for the treatment of mange mites in different animal species and primary myiasis caused by the New World screw worm fly, *Cochliomyia hominivorax*, and the Old World screw worm, *Chrysomya bezziana*, in dogs and cats [4–7]. The objective of the present study was to evaluate the effectiveness of sarolaner as part of the clinical management of furuncular myiasis in dogs naturally infested with *D. hominis* larvae.

Ten short-haired mixed-breed dogs with naturally occurring furuncular myiasis caused by *D. hominis* were enrolled in the study (Table 1). The dogs were housed in an animal shelter located in the municipality of Paty do Alferes, State of Rio Janeiro, southeastern Brazil. As inclusion criteria, dogs were presented with at least three furuncular myiasis lesions, and withdrawal time of 90 days from previous endectocide treatment was observed at the time of enrollment. Clinical diagnosis was achieved by the observation of skin nodules and visualization of larval motility through the lesion orifice. On physical examination of dogs, the number and body areas of furuncular myiasis lesions were registered. Sarolaner (Simparic™, Zoetis, Brazil) was administered orally to all dogs at a dose recommended by the manufacturer for flea and tick treatment and control (Table 1). All dogs were reexamined 24 h after treatment for the presence of lesions and for assessing the viability of larvae by visual inspection. Larvae were carefully removed from the nodules by digital compression, fixed in 70% alcohol and morphologically identified as *D. hominis* [8]. All dogs were examined one more time 30 days after sarolaner administration to follow up on the initially diagnosed lesions and assess for the potential presence of new ones.

**Table 1:** Signalment, sarolaner dose administered, affected body areas and number of lesions in ten dogs with furuncular myiasis caused by *Dermatobia hominis*

Dogs	Gender	Age (years)	Weight (kg)	Dose (mg/kg)	Number and body areas with furuncular myiasis lesions					
					TH	BE	LT	Limbs	Tail	Total
1	FS	3	11	3.6	0	2	5	0	0	7
2	FS	3	12	3.3	0	3	0	1	0	4
3	FS	6	18	2.2	0	0	9	0	1	10
4	FS	2	14	2.9	10	0	0	1	0	11
5	MN	4	14	2.9	0	0	2	1	0	3
6	FS	5	17	2.4	2	0	1	0	0	3
7	MN	6	16	2.5	0	0	3	0	2	5
8	FS	5	18	2.2	0	1	7	0	2	10
9	MN	4	16	2.5	2	0	14	0	0	16
10	MN	4	13	3.1	0	1	5	0	0	6

*FM* female spayed, *MN* male neutered, *TH* top of the head, *BE* base of the ears, *LT* lateral thorax

The number of furuncular myiasis lesions with a single larva ranged from 3 to 16 in the enrolled dogs. Signalment, sarolaner administered dose, number of lesions and affected body areas are presented in Table 1. The areas of the body presenting with furuncular myiasis varied in the affected dogs. The largest number of nodules was observed on the lateral thorax bilaterally (46/75; 61.4%) followed by the top of the head (14/75; 18.7%) and base of the ears (7/75; 9.4%). At 24 h post sarolaner administration, a total of 75 *D. hominis* larvae were retrieved from all 10 dogs. No live larvae were observed, demonstrating 100% larvicidal efficacy of sarolaner. No adverse events associated with the sarolaner treatment were noticed during the first 24 h post administration. Upon physical examination 30 days post-treatment with sarolaner, all previously observed furuncular myiasis lesions were healed and no new lesions were noticed in any of the dogs.

Furuncular myiasis by *D. hominis* larvae is of veterinary and medical concern due to the high incidence in companion animals, livestock and humans in Central and South America. Travel-associated human and animal dermatobiosis has been reported from North America and Europe, areas in which *D. hominis* is not endemic [9, 10].

Dogs with furuncular myiasis due to *D. hominis* larva infestation present with lesions characterized as erythematous draining nodules that cause intense discomfort. Infested human patients report pruritus, a sensation of movement and nocturnal stabbing pain within the lesions [1]. Treatment options to control the discomfort and the inflammatory reaction caused by the larva movement within the skin prior to its safe removal can be valuable in the clinical management and well-being of dogs with furuncular myiasis. In humans, the *D. hominis* lesions typically involve exposed areas of the body such as scalp, legs, arms and face [11]. Comparable situation has been previously reported in dogs where short-haired individuals tend to be more commonly affected [12]. The fact that all ten studied dogs were mixed breed dogs with a short hair coat further corroborates these observations. The body areas where the lesions are found, number and size of the lesions, as well as geographic origin and/or travel history of the affected dog should always be considered along with thorough physical and dermatological examinations.

In companion animals, different drug classes including macrocyclic lactones, spinosyns and neonicotinoids have been used in the clinical management and control of cutaneous myiasis [5, 7, 13, 14]. Recently, the off-label use of isoxazolines such as sarolaner and afoxolaner has been proven efficacious against myiasis caused by *C. hominivorax* in dogs [7, 14], lotilaner against *C. bezziana* [6], and fluralaner for treating *D. hominis* in cats [15]. Herein, we describe the use of oral sarolaner, an isoxazoline, in the clinical management of *D. hominis* larvae infestation at a standard dose recommended for the treatment and control of fleas and ticks. Isoxazolines are a novel class of ectoparasiticides that has unique characteristics of rapid absorption, prolonged duration, high margin of safety and broad-spectrum activity labeled for flea and tick treatment and prevention. Different drugs in this class are also used off label for the treatment of mange mites and myiasis.

In the present study, a 100% larvicidal efficacy of sarolaner was achieved within 24 h post-treatment, allowing dead, non-motile *D. hominis* larvae to be mechanically removed from all ten dogs by digital compression. The death of the larvae likely decreased the discomfort caused by their motility within the furuncular lesion and associated inflammation. Overall, the treatment also facilitated larval removal and the clinical management of the lesions, avoiding the need of sedation or a more invasive procedure on the skin. Moreover, dogs were reexamined 30 days after sarolaner administration, and not only all previously diagnosed lesions were healed, but new lesions were not observed. It is plausible that the monthly use of sarolaner for flea and tick prevention may also provide protection against the establishment of myiasis due to *D. hominis*. However, the efficacy of sarolaner against *D. hominis* first-stage larvae, which are deposited on the hosts’ skin by phoretic dipterans, remains to be clinically assessed.

## Conclusions

Oral administration of sarolaner at the dose recommended by the manufacturer seems to be effective for treating furuncular myiasis by *D. hominis* in dogs, reducing discomfort caused by the presence and movement of the larvae within the skin and facilitating removal of larvae.

## Data Availability

All data obtained in this study are included in this published article.

## Abbreviations

BE: Base of ear
CEUA: Ethics committee on the use of animals
LT: Lateral thorax
sp: Species
TH: Top of head
UFRRJ: Universidade Federal Rural do Rio de Janeiro
